# Synergy between* Rhizobium phaseoli* and* Acidithiobacillus ferrooxidans* in the Bioleaching Process of Copper

**DOI:** 10.1155/2016/9384767

**Published:** 2016-01-28

**Authors:** Xuecheng Zheng, Dongwei Li

**Affiliations:** ^1^College of Chemistry and Chemical Engineering, Southwest Petroleum University, Chengdu 610500, China; ^2^Oil and Gas Field Applied Chemistry Key Laboratory of Sichuan Province, Chengdu 610500, China; ^3^College of Resource and Environment Science, Chongqing University, Chongqing 400044, China

## Abstract

This study investigates the synergy of* Rhizobium phaseoli* and* Acidithiobacillus ferrooxidans* in the bioleaching process of copper. The results showed that additional* R. phaseoli* could increase leaching rate and cell number of* A. ferrooxidans*. When the initial cell number ratio between* A. ferrooxidans* and* R. phaseoli* was 2 : 1,* A. ferrooxidans* attained the highest final cell number of approximately 2 × 10^8^ cells/mL and the highest copper leaching rate of 29%, which is 7% higher than that in the group with* A. ferrooxidans* only.* R. phaseoli* may use metabolized polysaccharides from* A. ferrooxidans*, and organic acids could chelate or precipitate harmful heavy metals to reduce their damage on* A. ferrooxidans* and promote its growth. Organic acids could also damage the mineral lattice to increase the leaching effect.

## 1. Introduction

The environment and natural resources are important topics for research. Many researchers have reported the harmful effects of heavy metals from tailings reservoir on the environment [1, 2]. However, some heavy metals with high value, such as copper, could be recycled from the tailings. Bioleaching is commonly performed on low-grade copper because it is cheaper, more environment friendly, and more efficient than traditional methods [3, 4].

Bacteria require a strict reaction environment during the leaching process; thus, the low leaching rate and inefficiency have become a challenge [5]. Scholars have conducted studied electric fields [6], catalysts [7], metal cations [8], and other related topics to improve the leaching process. The use of synergy among different bacteria is an important approach in improving bioleaching. Donati et al. investigated the collaborative leaching of* Acidithiobacillus ferrooxidans* and* Acidithiobacillus thiooxidans* [9]. Deng et al. discussed the collaborative leaching of* Leptospirillum ferrooxidans* and* A. thiooxidans* [10, 11]. These studies found that mixed cultures demonstrate better performance than pure cultures. Zhu and Zhang found that the synergistic effects between chemoautotrophic bacteria and heterotrophic bacteria can improve the leaching rate of heavy metals [12]. During the leaching process, some additional bacteria could not endure the high concentration of heavy metals or the strong acidic condition. Few researchers have studied the assistance of high-tolerance chemoheterotrophic bacteria in the bioleaching process which could adapt to the environment at pH = 2. Thus, this study selected* Rhizobium phaseoli* to improve the leaching efficiency by using the synergy between* R. phaseoli* and* A. ferrooxidans*.* R. phaseoli* isolated from the nodules of kidney beans (a type of overaccumulated plant) could grow under strict conditions.

## 2. Materials and Methods

### 2.1. Materials

#### 2.1.1. Sample

**Figure 1:**
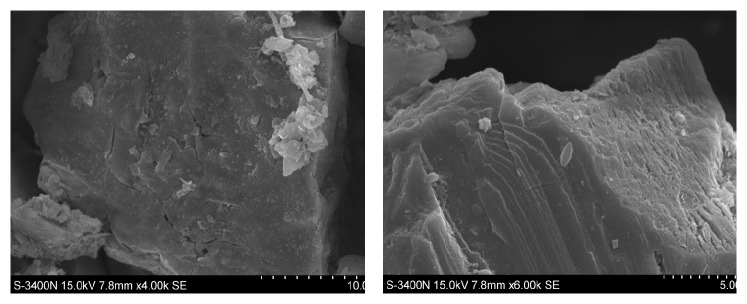
SEM pictures of tailing sample ((4000x) and (6000x)).

The tailing sample was collected from a copper mine reservoir in Yunnan province, China. Early sample analysis showed chalcopyrite as the main component, with 0.31% copper quality. However, the contents of other heavy metals especially toxic heavy metals (Cd 0.06403 mg/g, Pb 0.33251 mg/g, Ni 0.06227 mg/g, etc.) were too little to affect the bacteria, the average particle size of this sample was 18.30 *μ*m, and the content of sulfur was relatively high to provide the energy for* A. ferrooxidans*, so this tailing sample was suitable for bioleaching. The results of SEM testing and total content of heavy metals are listed in Figure 1.

#### 2.1.2. *A. ferrooxidans*

**Figure 2:**
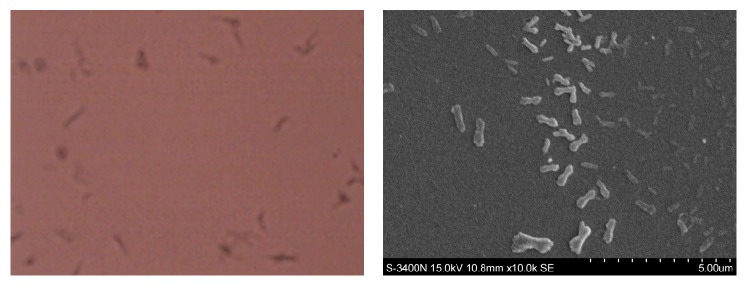
Pictures of* Acidithiobacillus ferrooxidans* under optical microscope (1000x) and SEM (10000x).

**Figure 3:**
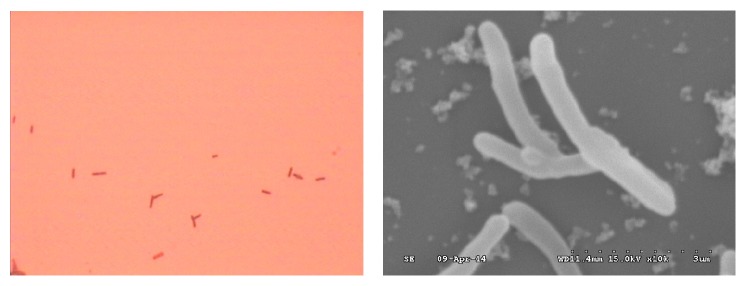
Pictures of* Rhizobium phaseoli* under optical microscope (1000x) and SEM (10000x).

The strain was isolated from an acid mine drainage and stored in the biological lab of Chongqing University, China. At the beginning of the experiment, 9K liquid medium was inoculated with the strain and then placed in constant temperature shaking with suitable environment. Only the bacteria in logarithmic phase were used for this experiment. Figures 2 and 3 show* A. ferrooxidans* under optical microscope and SEM, respectively. The pictures under optical microscope and SEM are listed in Figure 2.

#### 2.1.3. *R. phaseoli*

The strain, which is a type of heterotrophic and aerobic bacteria, was obtained from Agricultural Culture Collection of China and initially isolated from nodules of kidney bean. The strain could use many types of carbon source and grow in acidic environment. After previous domestication, the strain could grow normally in a copper concentration of 0.5 g/L and pH value of 2. The pictures under optical microscope and SEM are listed in Figure 3.

#### 2.1.4. Medium

The medium of* A. ferrooxidans* and* R. phaseoli* was, respectively, 9K liquid medium (composition: 3 g/L (NH_4_)_2_SO_4_, 0.5 g/L K_2_HPO_4_, 0.5 g/L MgSO_4_·7H_2_O, 0.01 g/L Ca(NO_3_)_2_, 0.23 g/L FeSO_4_·7H_2_O, 0.1 g/L KCl, and 1 L distilled water) and YMA liquid medium (yeast morphology agar, composition: 10 g/L mannitol, 1 g/L yeast powder, 0.5 g/L K_2_HPO_4_, 0.2 g/L MgSO_4_·7H_2_O, 0.1 g/L CaHPO_4_, 0.1 g/L NaCl, 4 mL 0.5% boric acid solution, 4 mL 0.5% sodium molybdate solution, 10 mL 0.4% Congo red, and 1 L distilled water).

#### 2.1.5. Experimental Equipment

Atomic fluorescence spectrometer (SK-2002B; Beijing, China), vertical pressure steam sterilizer (YXQ-LS-30S; Shanghai, China), constant temperature shaking (THZ-92A; Shanghai, China), pH-ORP tester (ORP-421; Shanghai, China), microscope (XSP-8C; Shanghai, China), thermostatic incubator (LRH-250-A; Shanghai, China), HPLC (Waters 2695; Shanghai, China), and hemocytometer (XB-R-25; Shanghai, China) were used in this experiment.

#### 2.1.6. Analytical Methods

The concentration of copper was tested with atomic fluorescence spectrometer, and leaching rate was defined as the copper concentration in leaching solution divided by the total copper content in the sample. We, respectively, dissolved 100 mg oxalic acid, 100 mg malic acid, 100 mg formic acid, 100 mg acetic acid, 100 mg succinate, 100 mg lactate, and 100 mg citrate with buffer solution to 50 mL, and then we detected organic acids with HPLC after filtration and ultrasonic degassing. The chromatographic conditions were listed as follows: chromatographic column was Hypersil BDS C_18_ (4.6 mm × 250 mm 5 *μ*m), 0.5% NH_4_H_2_PO_4_-H_3_PO_4_ buffer solution was the mobile phase, sample injection volume was 10 *μ*L, temperature was 30°C, flow rate was 1.0 mL/min, and measuring wavelength was 214 nm. We counted the number of bacteria by hemocytometer measurement. At first, we centrifuged or diluted the bacteria liquid until the concentration was at the order of appropriate magnitude and dyed the bacteria with trypan blue; after that we counted the bacteria in the grids for 3 times and calculated the concentration of bacteria with the corresponding formula.

### 2.2. Experimental Procedure

Tailing sample (10 g) was ground and divided into five groups, with each group containing three parallel test flasks. The first group was the sterile control group, and the other four groups were marked from A to D.* A. ferrooxidans* bacterial liquid (10 mL; 1.1 × 10^7^ cells/mL) was added to the four groups A–D. Groups B, C, and D were added with 10, 5, and 1 mL of* R. phaseoli* bacterial liquid (9 × 10^6^ cells/mL), respectively. The solution volumes were then adjusted to 100 mL, and noniron 9K liquid medium and initial pH values were adjusted to 2.2 with concentrated sulfuric acid included in all five groups. The initial* A. ferrooxidans*/*R. phaseoli* cell number ratios were about 1 : 1, 2 : 1, and 10 : 1 in groups B, C, and D, respectively, and tailing concentration was 10% (w/v) in each flask. Finally, all the five groups were placed in an air bath oscillator at 100 rpm and 25°C. The experiment lasted for 25 days, the pH values and cell numbers were measured daily, and copper concentrations were measured every 3 days. We calculated the mean values as the final testing results after omitting the finding with obvious errors.

## 3. Results and Discussion

### 3.1. Change of pH Values

**Figure 4:**
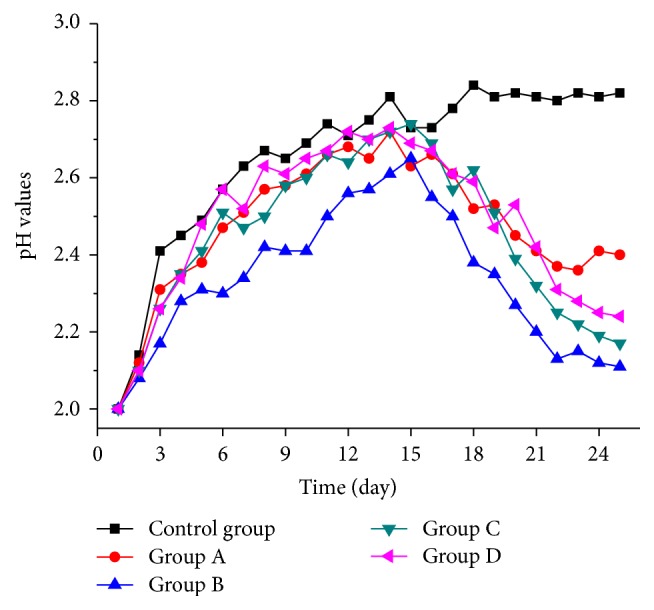
Change of pH values in 5 groups.

Figure 4 shows the changes in pH values among the five groups. The pH values in all the groups increased during the first 15 days. This increase was attributed to the reaction of some alkaline substances as chalcopyrite and ferrosulphide compounds with acids in the tailing and the proliferation of* A. ferrooxidans*, which also consumed hydrogen ions in the leaching solution, and the rate of neutralization reaction was much more than the rate of bacterial oxidation reaction. Furthermore, Fe^2+^ would be oxidized into Fe^3+^ as the energy of* A. ferrooxidans*; it consumed large number of hydrogen ions as follows:

(1) $$\begin{array}{c} 4{Fe}^{2+}+O_{2}+4H^{+}\overset{\text{bacteria}}{\to }4{Fe}^{3+}+2H_{2}O \end{array}$$

The pH level in the control group became stable on the 12th day and reached a value of 2.82 by the end of the experiment. The pH levels in the other groups started to decrease from the 16th day because of the near-depletion of alkaline substances and the hydrolysis reaction of Fe^3+^. With the growth of* A. ferrooxidans*, Fe^2+^ was oxidized to Fe^3+^, and the hydrolysis reaction of Fe^3+^ continuously advanced and generated numerous hydrogen ions [13]. By the end of the experiment, group B obtained the lowest pH value of 2.11 because more organic acids were secreted by* R. phaseoli* [14].

### 3.2. Test Results of Organic Acids

Organics, especially those with high content of small molecular organic acids, can cause the cytoplasm acidification of chemoautotrophic bacteria [15]. The reduction of oxygen at the extremity of the electron transport chain could also be affected. Thus, organics can damage the growth and metabolism of* A. ferrooxidans.* Organic acids were the main metabolites of* R. phaseoli.* We measured the contents of small molecular organic acids in groups B, C, and D with HPLC. The results are listed in Table 1.

As shown in Table 1, oxalic acid was the organic acid with the highest concentration (78.4 mg/L), whereas lactate was undetected. The concentration of formic acid, which is the most harmful acid to* A. ferrooxidans*, was relatively low [16]. Some studies showed that* A. ferrooxidans* can grow normally when the content of acetic acid or oxalic acid is ≤100 mg/L [17]. However, the proper content of organic acids can even stimulate the metabolic ability of Fe^2+^ in* A. ferrooxidans*.

### 3.3. Changes of the Cell Number of Bacteria

**Figure 5:**
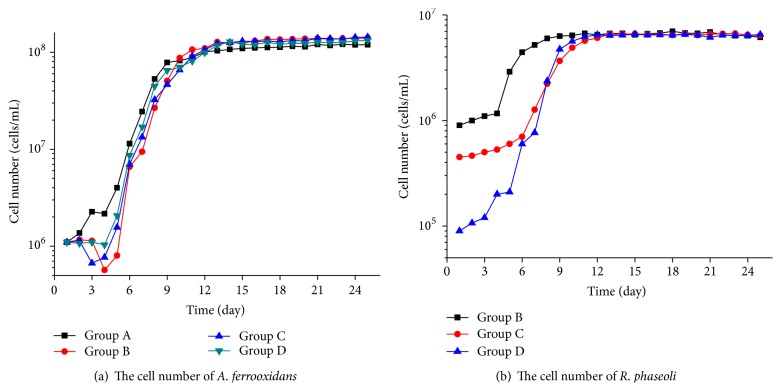
The cell number (ordinate after logarithmic transformation) of* Acidithiobacillus ferrooxidans* (a) and* Rhizobium phaseoli* (b).

Figure 5 shows the changes of the cell number of* R. phaseoli* and* A. ferrooxidans*.* R. phaseoli* could also grow normally without an added YMA liquid medium, and the remaining YMA medium in the previous bacteria liquid might provide the carbon source for* R. phaseoli.* However,* R. phaseoli* might have also used* A. ferrooxidans* metabolites as its carbon source. The logarithmic phase in group B with the most initial* R. phaseoli* was the shortest, and the stable phase in group B was also the earliest. In the last few days, the cell number of* R. phaseoli* in all the three groups tended to be stable and similar (about 5 × 10^6^ cells/mL) because the nutrients for* A. ferrooxidans* in the 9K medium added into the leaching environment were limited, and a certain number of* A. ferrooxidans* could provide limited organic compounds for* R. phaseoli*. Therefore, the differences became small toward the end of the experiment.

At the beginning of the experiment, the adaptation period of* A. ferrooxidans* in groups B, C, and D (with* R. phaseoli*) was evidently longer than that in group A (without* R. phaseoli*). This result may be attributed to the higher initial* R. phaseoli* concentration, which resulted in a longer adoption phase. The two reasons for this phenomenon are as follows. (1) The metabolites of* R. phaseoli* (mainly organic acids) inhibited the growth of* A. ferrooxidans* to some extent. (2)* R. phaseoli* competed with* A. ferrooxidans* for other sources, such as dissolved oxygen; thus,* A. ferrooxidans* needed more time to adapt to the new environment.

At the end of the experiment, the numbers of* A. ferrooxidans* in groups B, C, and D were, respectively, 1.4 × 10^8^ cells/mL, 1.43 × 10^8^ cells/mL, and 1.33 × 10^8^ cells/mL, which is about 2 × 10^7^ cells/mL more than 1.16 × 10^8^ cells/mL in group A. The main compositions of extracellular polymeric substances (EPS) of* A. ferrooxidans* were glucose, rhamnose, nucleic acid, and protein.* R. phaseoli* are chemoheterotrophic bacteria that consume metabolites in the EPS of* A. ferrooxidans* as complex organics were hydrolyzed into simple organics, ATP, and [H] under the action of catabolism enzyme.

The negative effect of organic acids on* A. ferrooxidans* could be reduced. The low concentration of organic acids could not harm* A. ferrooxidans* but could stimulate its growth. Organic acids released by* R. phaseoli* could chelate or precipitate the harmful heavy metals in the solution and could reduce the hazard to* A. ferrooxidans* [15].

### 3.4. Changes of the Leaching Rate of Copper

**Figure 6:**
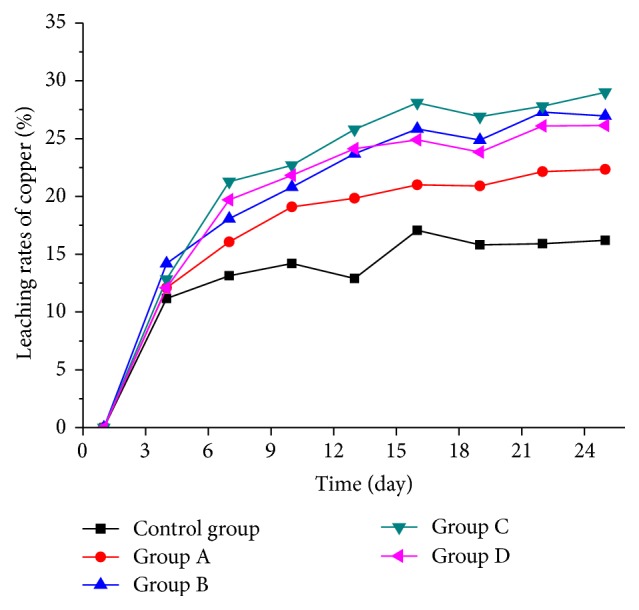
The changes of copper leaching rates.

Figure 6 shows the changes in copper leaching rates among the five groups. During the first four days, the differences among the five groups were not evident, and the leaching rates in groups with bacteria were obviously higher than that in the control group from the fourth day. The leaching rates in groups B, C, and D were higher than that in group A from the fourth day, as well. At the end of this experiment, the leaching rate of group A was 22.3%, which is 6.1% higher than that in the control group 16.2%, proving that* A. ferrooxidans* could promote copper leaching. Additional* R. phaseoli* could further raise the leaching rate to 29%, especially in group C, where the initial* A. ferrooxidans*/*R. phaseoli* cell number ratio was 2 : 1. At the 25th day, the leaching rates in groups B and D were 27% and 26.1%, respectively. The leaching results provided evidence that adding* R. phaseoli* could promote copper leaching further due to the reasons mentioned previously.

**Figure 7:**
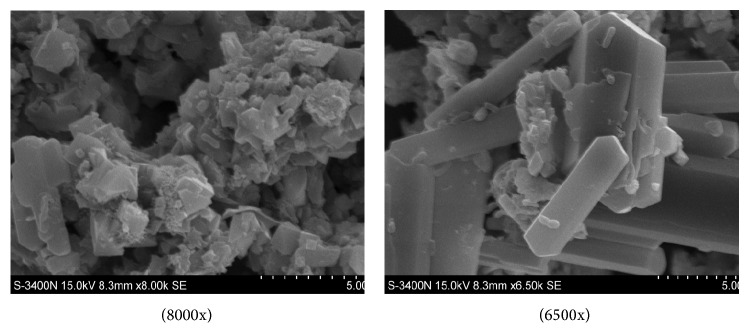
SEM pictures of tailing sample after leaching.

**Figure 8:**
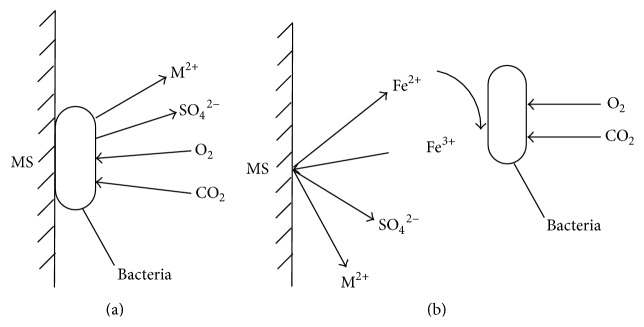
The schematic diagram of direct (a) and indirect leaching mechanism (b).

Figure 7 shows the tailing sample with bacteria on its surface in the leaching process.* A. ferrooxidans* adsorbed on the surface with its flagellum at the beginning. The bacteria oxidized minerals and obtained energy with the oxidizing enzymes of Fe^2+^ and S, and then the electrons released in the chemical oxidation reached the plasma membrane which was the combined point of bacterial respiration,* A. ferrooxidans* destroyed the surface lattices of metal sulfide minerals to oxidize them to the metal ions directly as mentioned earlier or oxidized Fe^2+^ into Fe^3+^, and then Fe^3+^ oxidized minerals with its strong oxidative activity. The direct and indirect mechanisms [18] are shown in Figure 8.

*R. phaseoli* could improve the growth and activity of* A. ferrooxidans*. Moreover,* R. phaseoli* mainly secreted organic acids, such as oxalic acid and citric acid. Oxalate anions contain strong and stable electrons, and each citric acid molecule can ionize three hydrogen ions. Thus, both oxalic acid and citric acid are strong organic acids that could damage mineral lattices to release copper ions into the leaching solution.

## 4. Conclusions

This study investigated the synergy between* R. phaseoli* and* A. ferrooxidans* in the bioleaching process. The cell number of* A. ferrooxidans* evidently increased because* R. phaseoli* consumed some of the organic metabolites of* A. ferrooxidans*. The number in group C was the highest at the beginning of the 8th day, and organic acids could chelate or precipitate harmful heavy metals to reduce their negative influence on* A. ferrooxidans*. Therefore, the cell number of* A. ferrooxidans* increased, whereas the cell number of* R. phaseoli* in the three groups tended to be stable and similar. The leaching results showed that* R. phaseoli* could obviously increase the copper leaching rate from 22% to 29%. In addition to the increase in the number of* A. ferrooxidans*, organic acids could also damage the mineral lattices to release copper ions into leaching solution. However, the low concentration of organic acids did not exceed the endurance limit of* A. ferrooxidans* and even promoted the leaching efficiency.

*Research Gap and Outlook*. This study found that the synergy between* Rhizobium phaseoli* and* Acidithiobacillus ferrooxidans* could promote copper leaching efficiency, but the leaching rate remained low and took a long time. For future work, the authors aim to investigate the catalyst for accelerating the leaching rates.

## Supplementary Material

The Supplementary Material contains the triplicate test data of the cell number of *Acidithiobacillus ferrooxidans* and *Rhizobium phaseoli* in FIGURE 5 and the changes of copper leaching rates in FIGURE 6.

## Figures and Tables

**Table 1:** Contents of organic acids in liquid culture mediums (mg/L).

Group	Oxalic acid	Malic acid	Formic acid	Acetic acid	Succinate	Lactate	Citrate
B	78.4	27.3	2.1	13.7	8.6	Not detected	36.9
C	74.2	31.1	1.7	10.9	7.9	Not detected	34.1
D	69.1	24.9	2.3	11.2	1.8	Not detected	31.8
